# TURBT versus RC in T1N0M0 non-urothelial bladder cancer: a population-based study

**DOI:** 10.3389/fonc.2026.1802962

**Published:** 2026-05-29

**Authors:** Qiuming He, Yuqi Chen, Tao Zhang, Wei Sheng, Ji Huang

**Affiliations:** 1 Jiangxi Cancer Hospital & Institute, Jiangxi Clinical Research Center for Cancer, The Second Affiliated Hospital of Nanchang Medical College, Nanchang, Jiangxi, China; 2 Jiangxi Medical College, Nanchang University, Nanchang, Jiangxi, China

**Keywords:** bladder cancer, histological subtype, NUBC, radical cystectomy, transurethral resection of bladder tumor

## Abstract

**Objectives:**

This study aims to compare overall survival (OS) and cancer-specific survival (CSS) between transurethral resection of bladder tumor (TURBT) and radical cystectomy (RC) in patients with T1N0M0 non-urothelial bladder cancer (NUBC). Additionally, it seeks to identify prognostic factors that can guide individualized treatment for this rare malignancy.

**Patients and methods:**

We conducted a retrospective analysis of patients with pathologically confirmed T1N0M0 NUBC using the SEER database (2004–2017). Patients were categorized by their most definitive surgical treatment: TURBT alone or RC. A 1:3 nearest-neighbor propensity score matching (PSM) without replacement was performed to balance baseline covariates between the two groups. OS and CSS were then compared between the matched cohorts.

**Results:**

Among 828 eligible patients (698 TURBT, 130 RC), significant differences in histology and treatment were observed. Following PSM, 496 patients (366 TURBT, 130 RC) were analyzed. The RC group demonstrated significantly superior 1-, 3-, and 5-year OS and CSS rates (OS: P<0.001; CSS: P=0.002). Multivariable Cox analysis identified neuroendocrine carcinoma, squamous cell carcinoma, and TURBT as independent risk factors for poorer prognosis, while RC was associated with improved survival (OS: HR=0.56, 95% CI 0.42–0.74, P<0.001; CSS: HR=0.48, 95% CI 0.32–0.72, P<0.001). Neuroendocrine and squamous cell carcinomas were associated with poorer OS than adenocarcinoma (P=0.002 and P<0.001, respectively). Married patients exhibited superior OS compared to single, divorced, widowed, or separated (SDWS) patients (P=0.003). Subgroup analyses generally favored RC, although survival differences were not statistically significant in specific subsets. For OS, these included patients aged <65 (P=0.120), male (P=0.071), low/intermediate grade (P=0.400), married (P=0.064), other histology (P=0.700), and SDWS (P=0.066). For CSS, these included patients aged <65 (P=0.120), low/intermediate grade (P=0.420), high grade (P=0.080), squamous cell carcinoma (P=0.170), other histology (P=0.690), SDWS (P=0.051), and non-first primary tumor (P=0.970).

**Conclusion:**

For patients with T1N0M0 NUBC, RC is associated with significantly improved survival outcomes compared to TURBT as definitive surgical management, with the greatest benefit observed in high-risk subtypes such as neuroendocrine and squamous cell carcinoma. However, this survival advantage must be carefully weighed against the substantial morbidity of RC and the potential for overtreatment. These findings support a risk-adapted, shared decision-making approach within a multidisciplinary framework, rather than a universal recommendation for RC in all T1N0M0 NUBC patients.

## Introduction

Bladder cancer represents a significant global health burden, ranking among the most common malignancies, with persistently high incidence and mortality rates. According to the 2021 U.S. cancer statistics, it is the fourth most common cancer in men and also shows significant prevalence in women, with notable disparities across geographic regions, races, and genders (1). Within this context, NUBC constitutes a rare and heterogeneous group of malignancies that are fundamentally distinct from urothelial carcinoma (UC), which accounts for 90–95% of all bladder cancers (2) (3). NUBC comprises approximately 5–10% of bladder cancer cases and encompasses diverse histological subtypes, each exhibiting unique biological behavior, epidemiological patterns, and clinical challenges. These subtypes are consistently associated with a significantly poorer prognosis than that of UC (4). This entity includes tumors originating from non-urothelial cell lineages (epithelial or mesenchymal) or UC with marked divergent differentiation (e.g., squamous or glandular), which alters their clinical behavior (5). Among NUBC subtypes, squamous cell carcinoma (SCC) is the most prevalent (2–5% of global bladder cancers), followed by adenocarcinoma (ADC, 0.5–2%), small-cell neuroendocrine carcinoma (SmCC, 0.5–1%), and rarer variants such as sarcomatoid and plasmacytoid carcinoma (3) (6). Although NUBC shares core symptoms with UC—such as painless gross hematuria, urinary frequency, and dysuria—it demonstrates more aggressive biology and often presents at advanced stages. Notably, 55% of NUBC cases are muscle-invasive (T3/T4) at diagnosis, compared to only 20% of UC cases (5). The clinical management of NUBC presents considerable challenges. Its low incidence has precluded large-scale prospective randomized trials, necessitating that current treatment strategies rely predominantly on retrospective studies, small case series, or extrapolations from UC management (7). While RC is regarded as the gold standard for localized NUBC, the 5-year overall survival rate remains suboptimal (8) (9). For non-muscle-invasive UC, TURBT remains the established standard. However, for non-muscle-invasive NUBC—specifically T1N0M0 disease—optimal management principles remain critically undefined. Although all histological variants are generally regarded as high-grade, high-risk tumors, data on the efficacy of intravesical therapies, such as Bacillus Calmette-Guérin (BCG) immunotherapy, in patients with variant histology are limited, with response rates potentially lower than those in pure UC (10). Consequently, a consensus on optimal management strategies remains elusive. Compounding this uncertainty, evidence suggests that NUBC patients experience a higher rate of pathological upstaging at subsequent radical surgery compared to UC patients, further jeopardizing their survival outcomes (11).

## Materials and methods

### Data source and study population

All patient data for this study were extracted from the Surveillance, Epidemiology, and End Results (SEER) database, which is maintained by the National Cancer Institute (NCI) (12). Data extraction and screening were conducted using SEER*Stat software (version 9.0.42.2), which accessed the Incidence - SEER Research Data, 17 Registries, November 2024 Sub database. SEER data are publicly available for research purposes, subject to permission.

Inclusion criteria included: 1) Diagnosis based on the International Classification of Diseases for Oncology, 3rd Edition (ICD-O-3) topography codes C67.0-C67.9; 2) Identification of four major non-urothelial histological variants of bladder cancer according to the 2016 World Health Organization (WHO) classification: SCC, adenocarcinoma, neuroendocrine carcinoma, and other epithelial tumors; 3) Age at diagnosis between 18 and 80 years. Exclusion criteria included: 1) Patients with lymph node or distant metastasis; 2) Patients with insufficient clinical or pathological data; 3) Patients with urachal carcinoma; 4) Cases identified solely by death certificate or autopsy; 5) Patients with unspecified surgical types.

**Figure 1:**
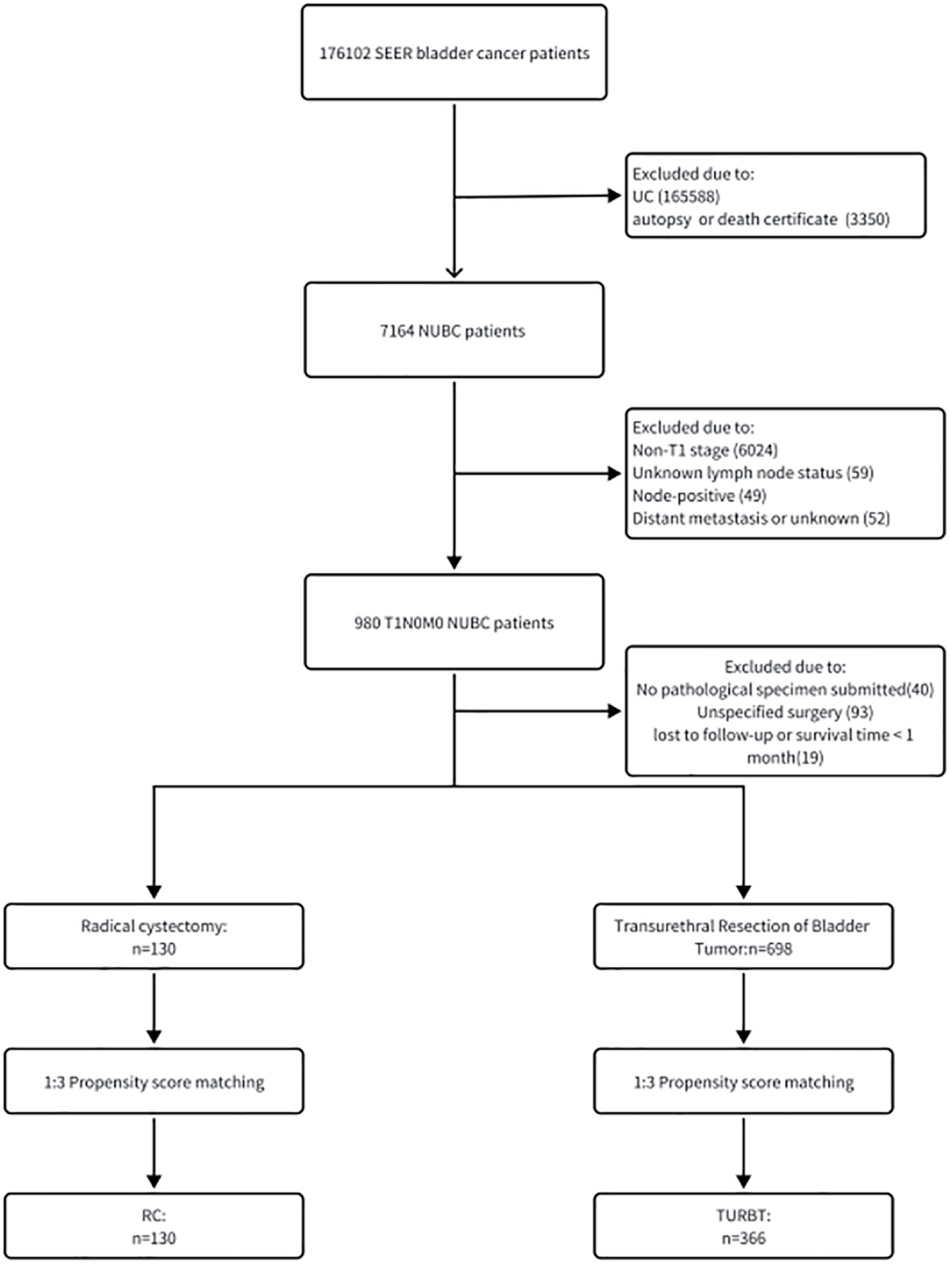
Flowchart of study cohort.

The initial dataset included variables such as Patient ID, Sex, Year of diagnosis, Year of follow-up recode, Age recode, Race recode (White, Black, Other), Marital status at diagnosis, Site recode ICD-O-3/WHO 2008, Primary Site, Behavior recode for analysis, Grade Recode (thru 2017), ICD-O-3 Hist/behav, malignant, Derived SEER Combined T (2016-2017), Derived SEER Combined N (2016-2017), Derived SEER Combined M (2016-2017), Derived AJCC T, 6th ed (2004-2015), Derived AJCC N, 6th ed (2004-2015), Derived AJCC M, 6th ed (2004-2015), RX Summ--Surg Prim Site (1998+), Radiation recode, Chemotherapy recode (yes, no/unk), COD to site recode, SEER cause-specific death classification, Survival months, Vital status recode (study cutoff used), First malignant primary indicator, and Total number of in situ/malignant tumors for each patient. Patients were classified into two comparison groups based on the most definitive surgical procedure recorded in the “RX Summ–Surg Prim Site” variable: those who underwent TURBT as their final surgical treatment (TURBT group) and those who underwent RC (RC group). This classification prevents contamination from patients who received diagnostic TURBT followed by definitive RC, as such patients would be coded under RC. A total of 828 patients diagnosed with T1N0M0 NUBC between 2004 and 2017 were initially identified for analysis (**Figure 1**).

### Ethical statement

This study utilized de-identified data from the SEER Program. Since the data are publicly available and anonymized, this research did not require approval from an Institutional Review Board (IRB) in accordance with SEER’s data use policy (https://seer.cancer.gov/data/use.html). The study was conducted in accordance with the principles outlined in the Declaration of Helsinki.

### Statistical analysis

Descriptive statistics included frequencies and proportions for categorical variables, which werecompared using the chi-square test. All statistical analyses were conducted using R and RStudio software (version 2025.9.2.418). To balance baseline characteristics and reduce selection bias, We performed a 1:3 propensity score matching using nearest-neighbor matching without replacement. Each patient in the RC group was matched to three patients in the TURBT group based on their propensity scores, thereby constructing a matched cohort comprising 366 TURBT patients and 130 RC patients. PSM was performed using the MatchIt package with a 1:3 nearest neighbor matching method. An additional file provides further details on this process [see **Additional File 1**]. Univariate and multivariable Cox proportional hazards regression models were utilized to identify independent prognostic factors. Kaplan-Meier survival curves were generated, and differences were assessed using the log-rank test. OS was defined as the duration from surgery to death from any cause or the last follow-up. CSS was defined as the duration from surgery to death from bladder cancer or the last contact (13). All statistical tests were conducted as two-sided, with a significance level set at P < 0.05.

## Results

### Demographic and clinicopathological characteristics

The initial study cohort consisted of 828 patients with confirmed T1N0M0 NUBC who met the inclusion and exclusion criteria. Prior to matching, this cohort included 698 patients who underwent TURBT and 130 patients who underwent RC. In the TURBT group, 34.2% of patients were under 65 years of age, 72.3% were male, 81.1% identified as White, and 57.0% were married. In contrast, in the RC group, 42.3% were under 65 years of age, 64.6% were male, 86.2% identified as White, and 61.5% were married. Concerning tumor characteristics, in the TURBT group, 24.8% had grade I/II tumors, 29.8% had adenocarcinoma, 67.0% had a first primary tumor, and 50.0% had a solitary tumor. In the RC group, 27.7% had grade I/II tumors, 32.3% had adenocarcinoma, 69.2% had a first primary tumor, and 46.9% had a solitary tumor. Regarding treatment, 93.6% of the TURBT group either did not receive radiotherapy or had an unknown radiotherapy status, while 78.8% either did not receive chemotherapy or had an unknown chemotherapy status. In the RC group, these proportions were 98.5% for radiotherapy and 67.7% for chemotherapy, respectively. Statistically significant differences (all P < 0.05) were observed between the two groups regarding the distribution of histological subtypes, radiotherapy, and chemotherapy.

To minimize confounding factors and more accurately reflect the intrinsic differences between the two treatment strategies, PSM was utilized. Following a 1:3 PSM adjustment for all potential confounders, the distributions of all included variables were adequately balanced between the two groups (**Table 1**). Ultimately, 366 patients who underwent TURBT were successfully matched with 130 patients who underwent RC, resulting in a matched sample of 496 patients for subsequent comparative effectiveness analysis.

**Table 1:** Demographic and clinical pathological characteristics distribution of bladder cancer patients treated with TURBT and RC before and after matching.

Variables	Groups, N (%)					
	Crude dataset			Matched dataset		
	TURBT (= 698)	RC (=130)	P	TURBT (=366)	RC (=130)	P
Age						
<65	239(34.2%)	55(42.3%)	0.096	149(40.7%)	55(42.3%)	0.830
≥65	459(65.8%)	75(57.7%)		217(59.3%)	75(57.7%)	
Sex						
Female	193(27.7%)	46(35.4%)	0.093	115(31.4%)	46(35.4%)	0.471
Male	505(72.3%)	84(64.6%)		251(68.6%)	84(64.6%)	
Race						
White	566(81.1%)	112(86.2%)	0.346	320(87.4%)	112(86.2%)	0.549
Black	89(12.8%)	11(8.5%)		34(9.3%)	11(8.5%)	
Other	43(6.2%)	7(5.4%)		12(3.3%)	7(5.4%)	
Marital status						
Married	398(57.0%)	80(61.5%)	0.588	218(59.6%)	80(61.5%)	0.868
SDWS	245(35.1%)	42(32.3%)		121(33.1%)	42(32.3%)	
Unknown	55(7.9%)	8(6.2%)		27(7.4%)	8(6.2%)	
Grade						
I/II	173(24.8%)	36(27.7%)	0.547	96(26.2%)	36(27.7%)	0.949
III/IV	339(48.6%)	65(50.0%)		187(51.1%)	65(50.0%)	
Unknown	186(26.6%)	29(22.3%)		83(22.7%)	29(22.3%)	
Histological subtypes						
Adenocarcinoma	208(29.8%)	42(32.3%)	0.005	131(35.8%)	42(32.3%)	0.311
Neuroendocrine carcinoma	109(15.6%)	29(22.3%)		64(17.5%)	29(22.3%)	
Other	234(33.5%)	24(18.5%)		88(24.0%)	24(18.5%)	
Squamous carcinoma	147(21.1%)	35(26.9%)		83(22.7%)	35(26.9%)	
Radiation therapy						
None/Unknown	653(93.6%)	128(98.5%)	0.044	359(98.1%)	128(98.5%)	1.000
Yes	45(6.4%)	2(1.5%)		7(1.9%)	2(1.5%)	
Chemotherapy						
No/Unknown	550(78.8%)	88(67.7%)	0.008	259(70.8%)	88(67.7%)	0.586
Yes	148(21.2%)	42(32.3%)		107(29.2%)	42(32.3%)	
First primary *in situ* tumor						
No	230(33.0%)	40(30.8%)	0.700	113(30.9%)	40(30.8%)	1.000
Yes	468(67.0%)	90(69.2%)		253(69.1%)	90(69.2%)	
Number of Tumors						
Solitary tumor	349(50.0%)	61(46.9%)	0.583	167(45.6%)	61(46.9%)	0.879
Multiple tumors	349(50.0%)	69(53.1%)		199(54.4%)	69(53.1%)	

### Survival and prognostic analysis in the matched cohort

**Figure 2:**
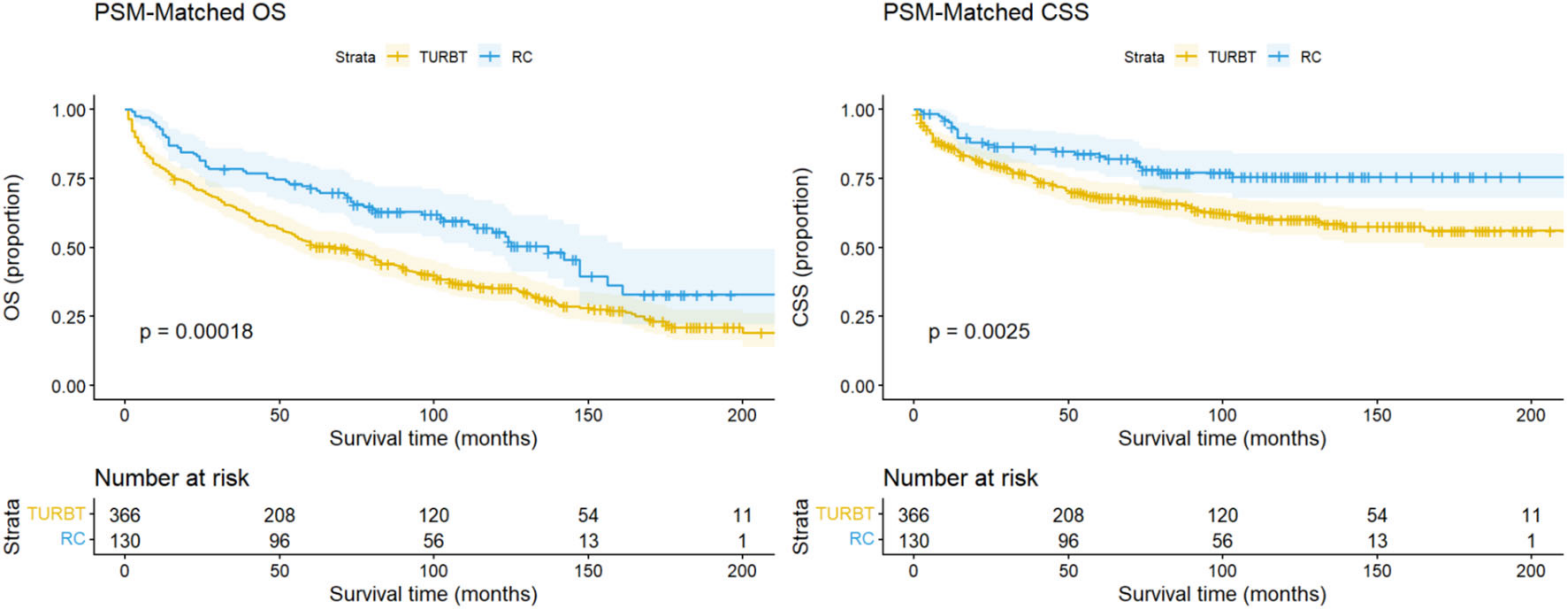
OS and CSS by surgical modality in propensity-matched patients.

In the matched population, the OS rates at 1, 3, and 5 years were 81.7%, 67.7%, and 56.3%, respectively. The corresponding CSS rates were 88.3%, 79.1%, and 72.0%. Further analysis revealed that the RC group had significantly superior survival outcomes (**Figure 2**). Specifically, the RC group demonstrated OS rates of 90.8%, 78.5%, and 71.5% at 1, 3, and 5 years, respectively, compared to 78.4%, 63.9%, and 51.0% in the TURBT group. Similarly, for CSS, the RC group exhibited rates of 93.8%, 86.5%, and 83.0%, which were significantly higher than the 86.3%, 76.4%, and 67.9% observed in the TURBT group.

Prognostic factors for OS and CSS in T1N0M0 NUBC patients were evaluated (**Table 2**). The results indicated that neuroendocrine carcinoma and squamous cell carcinoma were independently associated with poorer OS and CSS outcomes. Age ≥ 65 years was independently associated with poorer OS, whereas married status and the presence of a first primary tumor were associated with improved OS. Furthermore, compared to TURBT, RC was significantly associated with favorable survival outcomes (OS: HR=0.56, 95% CI 0.42–0.74, P < 0.001; CSS: HR=0.48, 95% CI 0.32–0.72, P < 0.001).

**Table 2:** Univariable and multivariable Cox regression analyses for OS and CSS in the matched cohort.

Variables	Overall survival			Cancer-specific survival		
	Univariate P	Multivariate P	Hazard ratio (95% CI)	Univariate P	Multivariate P	Hazard ratio (95% CI)
Age						
<65	Ref.	Ref.	Ref.	Ref.	Ref.	Ref.
≥65	<0.001	<0.001	1.61 (1.26-2.05)	0.027	0.077	1.35 (0.97-1.88)
Sex						
Female	Ref.			Ref.		
Male	0.455			0.393		
Race						
White	Ref.			Ref.		
Black	0.527			0.278		
Other	0.708			0.763		
Marital status						
Married	Ref.	Ref.		Ref.		
SDWS	0.011	0.003	0.69 (0.55-0.88)	0.063		
Unknown	0.556	0.437	1.18 (0.77-1.81)	0.941		
Grade						
I/II	Ref.			Ref.		
III/IV	0.693			0.180		
Unknown	0.382			0.938		
Histological subtypes						
Adenocarcinoma	Ref.	Ref.	Ref.	Ref.	Ref.	Ref.
Neuroendocrine carcinoma	0.018	0.002	1.71 (1.22-2.39)	0.011	0.005	1.91 (1.22-3.01)
Other	0.326	0.154	1.25 (0.92-1.70)	0.957	0.874	0.96 (0.60-1.55)
Squamous carcinoma	<0.001	<0.001	1.82 (1.36-2.44)	<0.001	<0.001	2.11 (1.39-3.19)
Radiation therapy						
None/Unknown	Ref.			Ref.		
Yes	0.010			0.031		
Chemotherapy						
No/Unknown	Ref.			Ref.		
Yes	0.585			0.072		
First primary *in situ* tumor						
No	Ref.	Ref.	Ref.	Ref.		
Yes	<0.001	0.020	0.74 (0.58-0.95)	0.093		
Number of Tumors						
Solitary tumor	Ref.			Ref.		
Multiple tumors	0.068			0.669		
Surgery						
TURBT	Ref.	Ref.	Ref.	Ref.	Ref.	Ref.
RC	<0.001	<0.001	0.56 (0.42-0.74)	0.003	<0.001	0.48 (032-0.72)

The small sample size in the radiotherapy group necessitates that the results from the univariable Cox regression be interpreted with caution; therefore, this variable was not included in the multivariable Cox regression analysis.

### Subgroup interaction analysis

**Figure 3:**
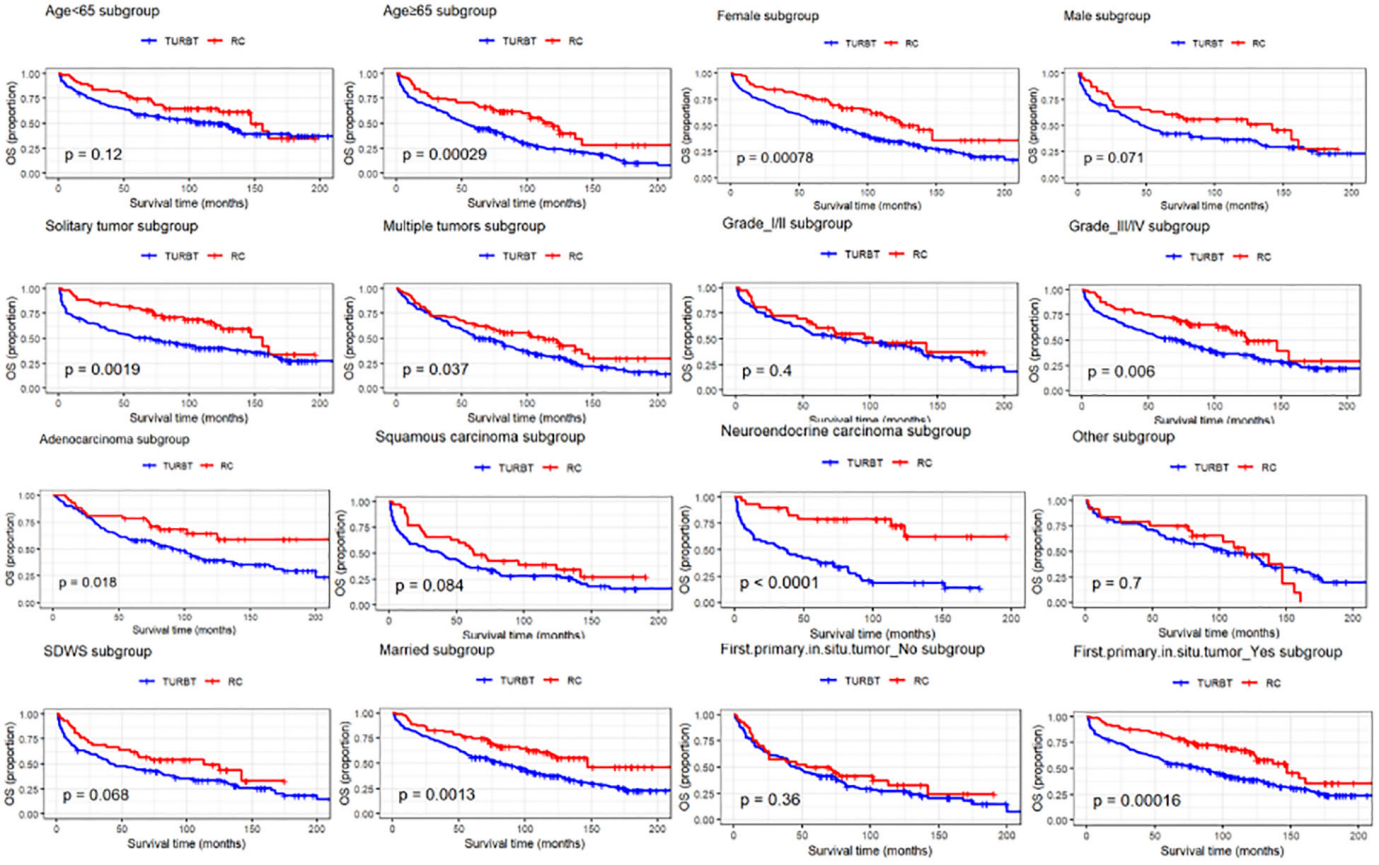
Kaplan-Meier curves for overall survival based on clinical characteristics.

**Figure 4:**
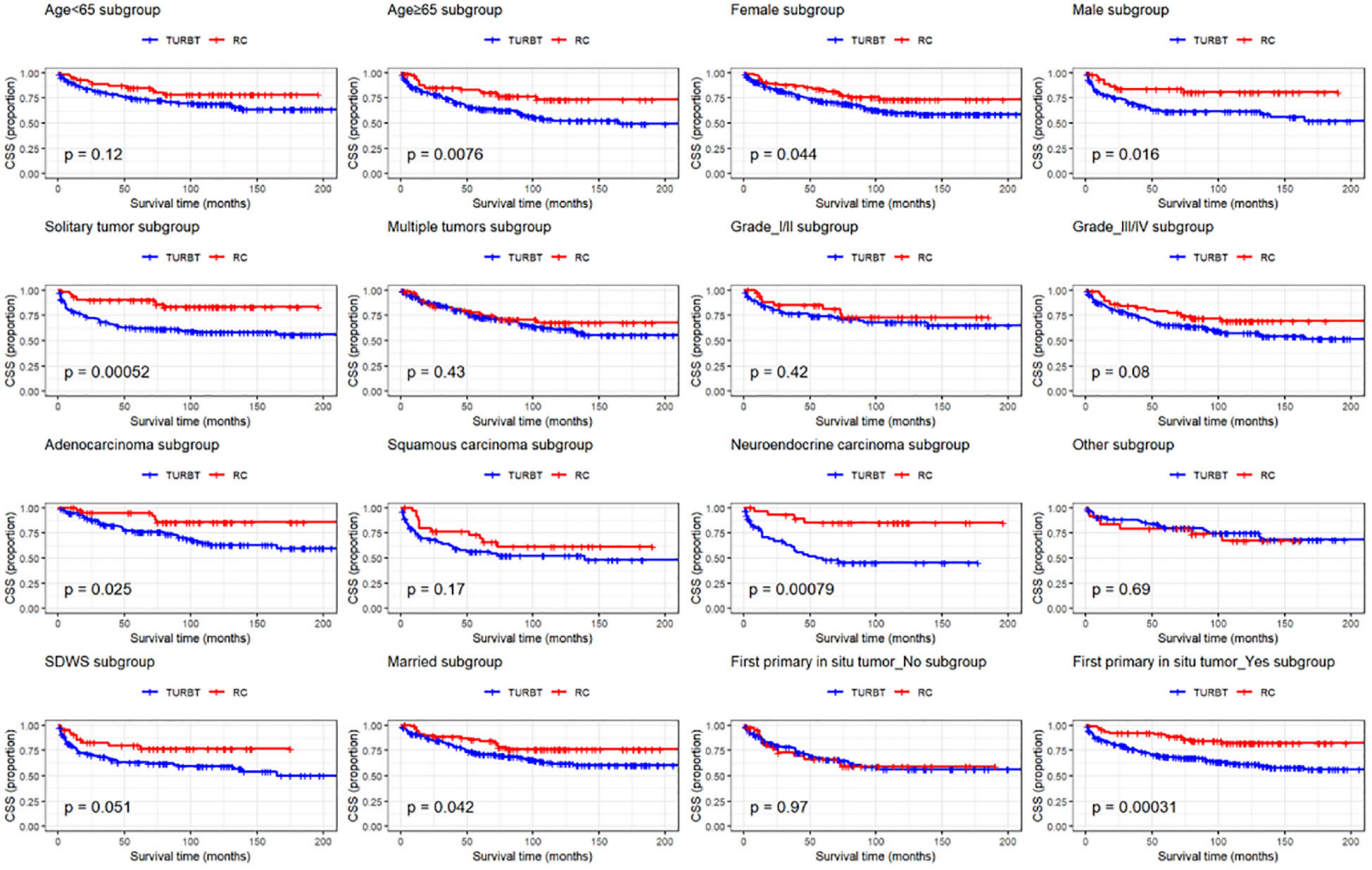
Kaplan-Meier curves for cancer-specific survival based on clinical characteristics.

Subgroup analyses were conducted to examine the differential treatment effects across various clinicopathological characteristics. The analysis revealed that RC was generally associated with better OS and CSS outcomes than TURBT in most subgroups. However, no significant difference in OS was observed in the following subgroups: age < 65 years (P=0.120), male (P=0.071), low/intermediate grade (P=0.400), married status (P=0.064), other histology (P=0.700), and SDWS status (P=0.066) (**Figure 3**). Similarly, no significant difference in CSS was found in the following subgroups: age < 65 years (P=0.120), low/intermediate grade (P=0.420), high grade (P=0.080), squamous cell carcinoma (P=0.170), other histology (P=0.690), SDWS status (P=0.051), and non-first primary tumor status (P=0.970) (**Figure 4**).

**Figure 5:**
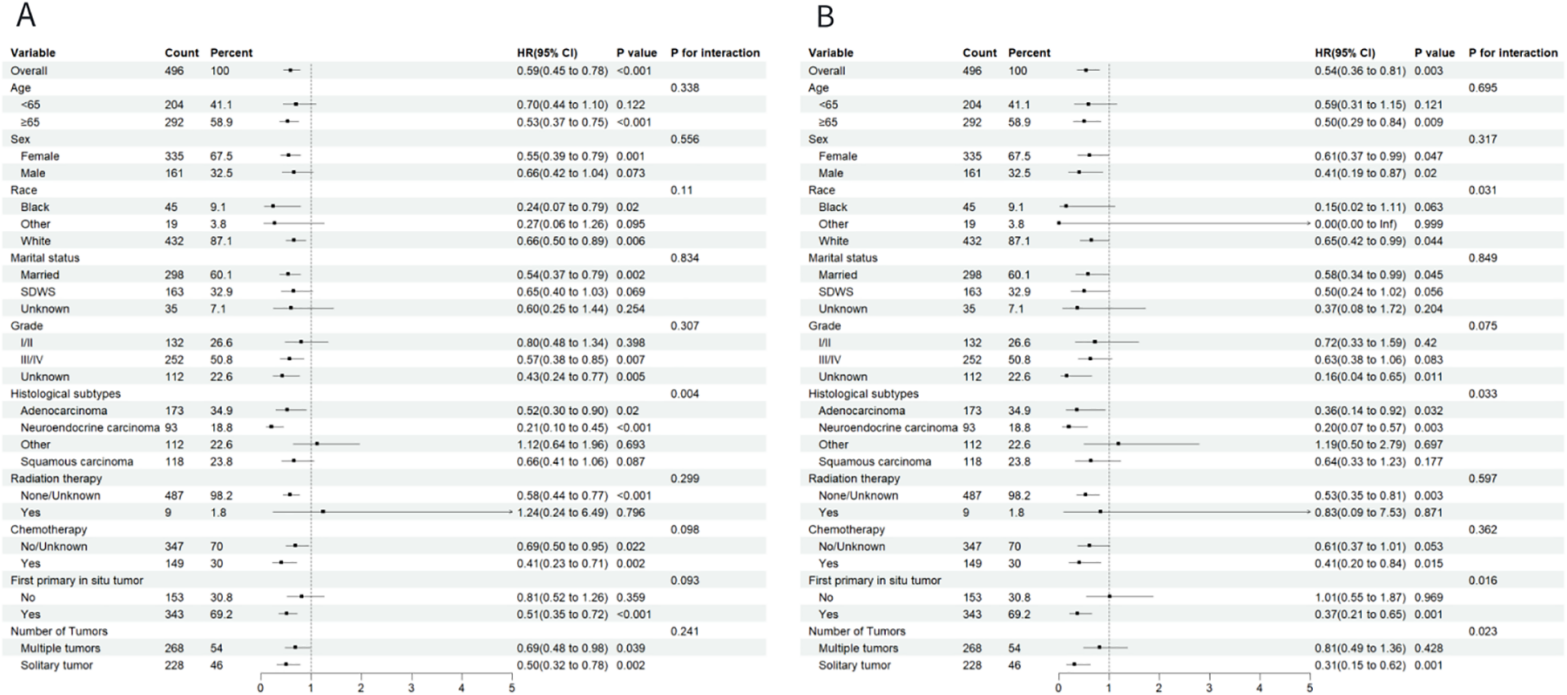
Forest plot of the association between surgical modality and overall mortality and cancer-specific mortality in subgroup analyses. **(A)** Overall Survival. **(B)** Cancer-Specific Survival.

Furthermore, forest plots illustrated the hazard ratios (HRs) and interactions for various subgroups in the OS and CSS analyses (**Figure 5**). In the OS analysis, the overall analysis indicated that the exposure factor (RC vs. TURBT) was associated with a reduced risk of the outcome (HR=0.58, 95% CI 0.44–0.77, P < 0.001). Significant heterogeneity in effect was observed only for histological subtype (P for interaction = 0.004), while effect consistency was maintained across other subgroups. In the CSS analysis, the overall results also demonstrated a significant risk reduction associated with RC (HR=0.54, 95% CI 0.36–0.81, P=0.003). However, significant interactions were identified for race (P for interaction = 0.031), histological subtype (P=0.033), first primary tumor status (P=0.016), and tumor number subgroup (P=0.023).

## Discussion

Our analysis of patients with T1N0M0 NUBC from the SEER database provides novel insights into this exceedingly rare entity. The primary finding indicates that after balancing confounders using PSM, patients undergoing RC exhibited significantly superior OS and CSS compared to those treated with TURBT. However, subgroup analyses revealed scenarios in which this difference was not statistically significant. This underscores the critical need for individualized treatment decision-making for this specific patient population. A core strength of this study lies in the implementation of a robust statistical strategy to control for confounding variables. By employing PSM, we effectively balanced the baseline distribution of potential confounders between the TURBT and RC groups, significantly mitigating selection bias and enhancing the reliability of survival outcome comparisons.

The investigation of prognostic factors within the matched cohort provides essential insights for customizing treatment strategies for this specific subtype of bladder cancer, highlighting the variability in patient prognosis across various clinicopathological characteristics. Specifically, neuroendocrine carcinoma and squamous cell carcinoma were independently linked to poorer OS and CSS. These findings are consistent with studies utilizing the National Cancer Database and California Cancer Registry, which consistently report lower overall and disease-specific survival rates for NUBC patients compared to those with urothelial carcinoma (8, 14). Our results support existing literature indicating that squamous cell carcinoma and small-cell neuroendocrine carcinoma exhibit the most aggressive natural history across all stages (4, 15). The age of 65 was selected as the stratification threshold based on the eligibility criteria for the U.S. Medicare program. This is supported by epidemiological data demonstrating that the incidence of bladder cancer increases with age, peaking at approximately 65 years (16). Notably, our analysis identified age ≥65 years as an independent predictor of poorer OS, while married status and being a first primary tumor were linked to improved OS (though not significantly for CSS). The survival advantage observed in married patients may be attributed to increased social support, which can positively influence treatment adherence and psychological well-being (17). Moreover, our finding regarding the prognostic disadvantage of non-first primary tumors aligns with a large-scale study by Wang X et al., which demonstrated significantly poorer outcomes for patients with second primary cancers compared to those with first primary cancers (18).

The subgroup analyses highlight the complexity involved in selecting the optimal treatment for T1N0M0 NUBC. While RC was generally associated with better survival rates across most subgroups, exceptions were noted. For instance, no significant difference in OS was observed in subgroups defined by age <65 years, male sex, low/intermediate tumor grade, married status, other histologies, or SDWS status. Similarly, CSS differences were not statistically significant in subgroups including age <65 years, low/intermediate or high grade, squamous cell carcinoma histology, other histologies, SDWS status, and non-first primary tumors. These findings are critical for clinical translation, as they identify patient subsets for whom the oncologic imperative for immediate RC may be less compelling, thereby opening the door for informed discussions about bladder-sparing approaches. Furthermore, the significant interaction effects observed for histological subtype in the OS analysis, as well as for race, histological subtype, first primary tumor status, and tumor number in the CSS analysis, further emphasize the necessity of integrating multi-dimensional clinical and potentially molecular factors to accurately predict survival outcomes and guide individualized treatment planning for T1N0M0 NUBC patients.

Recent advancements in risk stratification for T1 bladder cancer further highlight the complexity of treatment decision-making in early-stage non-urothelial carcinoma. A comprehensive systematic review and meta-analysis conducted by Piccolini et al. demonstrated that micrometric substaging (T1m/T1e, 0.5mm cutoff) offers superior prognostic discrimination for both recurrence and progression compared to traditional histoanatomical substaging based on muscularis mucosae invasion in T1 non-muscle-invasive bladder cancer (19). Notably, while histoanatomical substaging was significantly associated with disease progression (HR=2.65, 95% CI 1.79–3.92, p < 0.001), it failed to predict recurrence (HR=1.19, 95% CI 0.88–1.61, p = 0.3), whereas micrometric substaging correlated with both outcomes. These findings underscore the substantial heterogeneity within T1 disease and the critical need for standardized, objective prognostic parameters—an imperative that extends directly to T1N0M0 NUBC. Given that NUBC subtypes, such as small-cell neuroendocrine carcinoma and squamous cell carcinoma, exhibit even more aggressive biological behavior than urothelial carcinoma, the development and validation of subtype-specific substaging or molecular stratification systems (e.g., integrating TP53, PD-L1, or FGFR3 status) may be essential for refining treatment algorithms. The observation by Piccolini et al. that en bloc resection improves T1 substaging accuracy further supports the potential oncological superiority of radical cystectomy over transurethral resection in achieving precise risk stratification for NUBC, where accurate assessment of lamina propria invasion depth and margin status is paramount. Nevertheless, the lack of international consensus on optimal substaging methods, as emphasized in that study, reflects the current absence of standardized treatment guidelines for T1N0M0 NUBC and reinforces the necessity for individualized, multidisciplinary decision-making until prospective, subtype-specific evidence becomes available.

Currently, RC remains the gold standard for localized NUBC, demonstrating a superior survival benefit compared to TURBT. Multicenter and population-based studies consistently indicate that RC confers a survival advantage for major NUBC subtypes, including squamous cell carcinoma, adenocarcinoma, and small-cell carcinoma (20). Furthermore, propensity score-matched analyses have confirmed that the survival benefit of RC is not merely an artifact of patient selection bias, but rather reflects a genuine oncological superiority (21).

NUBC is a heterogeneous disease entity with clinical behaviors distinct from those of urothelial carcinoma (5). Its histological variants present significant diagnostic and management challenges, necessitating dedicated research and individualized clinical strategies. These subtypes frequently exhibit highly aggressive biological behavior, resulting in poor prognosis and lower response rates to conventional therapies. They are also more prevalent among younger patient populations, progress rapidly, and are often driven by specific molecular events (e.g., TP53 mutations, FGFR3 alterations, or ERBB2 amplification) (22). Consequently, there is an urgent need for prospective trials to validate the differences in efficacy between TURBT and RC in subtype-specific cohorts, particularly for T1N0M0 NUBC. Future research should also focus on developing predictive models that integrate clinical factors (e.g., tumor size, subtype, and stage) with molecular biomarkers (e.g., TP53 and PD-L1) to guide personalized surgical decision-making, aiming for an optimal balance between oncologic efficacy and quality of life (23).

It is essential to emphasize that, while our findings offer new insights for this patient population, the study is not without limitations. The most significant limitation, inherent to all analyses of the SEER database, is the lack of detailed information on patient performance status, comorbidities, and smoking history. This introduces the potential for ‘healthy candidate bias,’ as patients selected for radical cystectomy are likely inherently healthier and possess a greater physiological reserve to tolerate major surgery. Consequently, the observed survival advantage of RC may be partially attributable to patient selection rather than the procedure itself. Although we employed rigorous PSM to balance all measurable confounders and included marital status as a proxy for social support, the impact of residual, unmeasured confounding cannot be excluded. Our findings should therefore be interpreted as hypothesis-generating. Furthermore, the SEER database lacks specific details regarding chemotherapy or radiotherapy regimens, doses, and durations, limiting our ability to analyze the clinical value of adjuvant therapies. Another limitation is the absence of data on intravesical agents, which are a cornerstone of bladder-sparing management for T1 disease. We cannot ascertain the proportion of patients in the TURBT group who received adjuvant intravesical BCG or chemotherapy. However, current literature suggests that non-urothelial variants may exhibit limited responsiveness to standard intravesical therapies, potentially mitigating the impact of this missing variable on our primary comparison, though its true effect remains unknown (10). Furthermore, the absence of tumor size limits further stratification of the TURBT group by this clinically relevant parameter, which may influence decisions regarding early cystectomy. The absence of molecular markers relevant to NUBC prognosis and treatment response also restricts insights into precision medicine. Additionally, it is crucial to note that the TURBT group in our study represents patients for whom TURBT was the final surgical treatment. The inferior outcomes observed may reflect the aggressive biology of T1 NUBC managed with a bladder-sparing approach, which can lead to under-staging and subsequent progression, rather than TURBT being inferior as a diagnostic or staging tool. Despite these limitations, our study offers valuable perspectives on the management of this specific and challenging bladder cancer subtype by providing real-world, population-level evidence.

In translating our findings to clinical practice, the significant quality-of-life implications of RC must be placed at the forefront of decision-making. RC entails permanent urinary diversion, substantial sexual dysfunction, and considerable perioperative morbidity. Thus, the survival benefit we observed, particularly in lower-risk subgroups, must be carefully weighed against these life-altering consequences. Our results are not a universal endorsement of RC for all T1N0M0 NUBC patients, but rather a call for its strong consideration in high-risk variants (e.g., neuroendocrine and squamous cell carcinoma) within a multidisciplinary team framework that fully integrates patient values and preferences.

## Conclusion

In summary, for patients with T1N0M0 NUBC, RC is associated with significantly improved survival outcomes compared to TURBT as definitive surgical management, with survival benefits most pronounced in high-risk histological subtypes such as neuroendocrine and squamous cell carcinoma. However, this survival advantage must be carefully weighed against the substantial morbidity, quality-of-life impact, and potential for overtreatment associated with RC. Our findings are best applied to inform a multidisciplinary, shared decision-making process, guiding individualized treatment strategies that balance the imperative of oncologic control against organ preservation and patient values. The statistically non-significant survival differences observed in several patient subgroups underscore potential opportunities for selective bladder-sparing approaches. Future prospective, molecularly-driven studies are critical to refine risk stratification, enabling the identification of patients who can be safely managed with bladder preservation and those for whom upfront RC is the strongly preferred approach.

## Data Availability

Publicly available datasets were analyzed in this study. This data can be found here: https://seer.cancer.gov/.

## Supplementary material

Additional File 1 Covariate Balance Plot Before and After PSM Before and After Matching.
