# Supplementary material for: TURBT versus RC in T1N0M0 non-urothelial bladder cancer: a population-based study
Source: Front Oncol. 2026 May 29;16:1802962. doi: 10.3389/fonc.2026.1802962 (PMC13259815; doi:10.3389/fonc.2026.1802962)

**Supplementary figure 1:** **Covariate balance plot: Clinical covariate mean differences (unadjusted vs adjusted samples)**


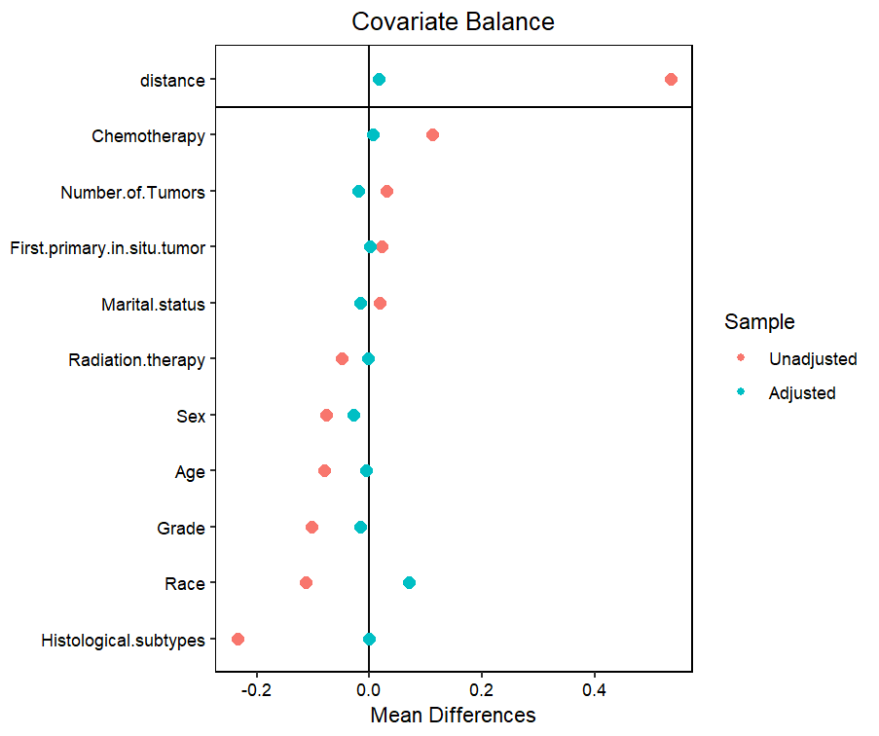


**Supplementary figure 1:Propensity score distribution: TURBT vs RC groups (pre- vs post-PSM)**
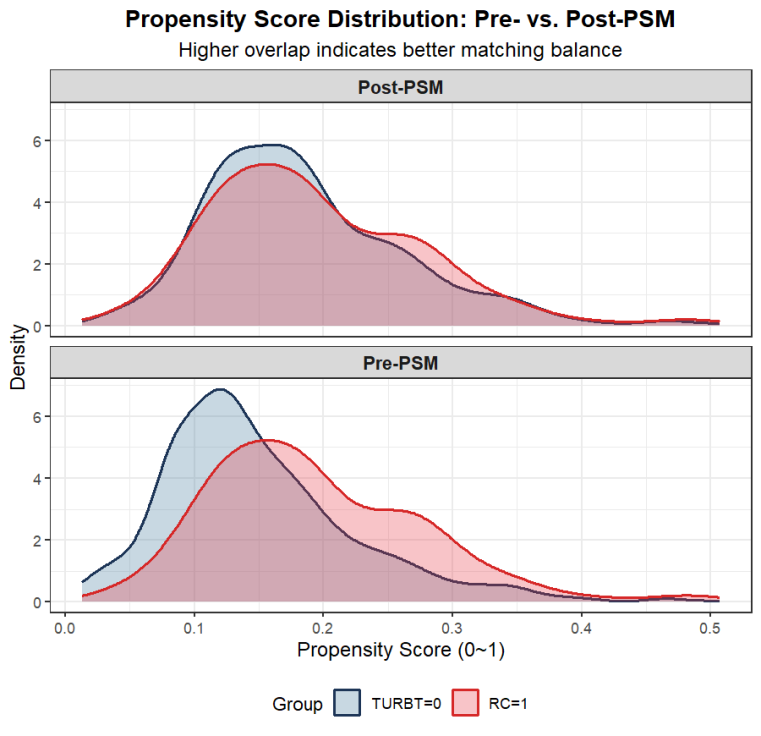

Supplement: Additional File 1 — Covariate Balance Plot Before and After PSM Before and After Matching. [file DataSheet1.docx]
